# Range expansion can promote the evolution of plastic generalism in coarse-grained landscapes

**DOI:** 10.1093/evlett/qrad062

**Published:** 2023-12-14

**Authors:** Caitlin M Miller, Jeremy A Draghi

**Affiliations:** Department of Biological Sciences, Virginia Polytechnic Institute and State University, Blacksburg, VA, United States; Department of Biological Sciences, Virginia Polytechnic Institute and State University, Blacksburg, VA, United States

**Keywords:** range expansion, phenotypic plasticity, complex adaptation, pleiotropy, constraints

## Abstract

Phenotypic plasticity is one way for organisms to deal with variable environments through generalism. However, plasticity is not found universally and its evolution may be constrained by costs and other limitations such as complexity: the need for multiple mutational steps before the adaptation is realized. Theory predicts that greater experienced heterogeneity, such as organisms may encounter when spatial heterogeneity is fine-grained relative to dispersal, should favor the evolution of a broader niche. Here we tested this prediction via simulation. We found that, contrary to classical predictions, coarse-grained landscapes can be the most favorable for the evolution of plasticity, but only when populations encounter those landscapes through range expansion. During these range expansions, coarse-grained landscapes select for each step in the complex mutational pathway to plastic generalism by blocking the dispersal of specialists. These circumstances provide ecological opportunities for innovative mutations that change the niche. Our results indicate a new mechanism by which range expansion and spatially structured landscapes interact to shape evolution and reveal that the environments in which a complex adaptation has the highest fitness may not be the most favorable for its evolution.

## Teaser

What types of environments favor specialists and which drive the evolution of generalists? Intuitively, generalists should do best when landscapes are highly variable. However, in simulations we found that coarse-grained landscapes, featuring large clusters of each type of environment, could be the most favorable for the evolution of plastic generalists. This surprising result occurred in populations expanding into new areas: in this scenario, coarse-grained heterogeneity was both selected for generalists and impeded the movement of specialists. In this study, we show that barriers to the dispersal of specialists can reward mutations that change an organism’s niche. This interaction of ecology and evolution drives the exploration of genotype space, rapidly producing the complex trait of plasticity. Our results point to surprising ways in which expanding populations experience selection differently from those that are stationary, potentially leading to innovative evolutionary outcomes.

## Introduction

Many organisms are experiencing new and rapid changes to their environments due to climate change and anthropogenic disturbances, such as habitat fragmentation and introduction of non-native species (Matesanz et al., 2010). Changing climatic conditions can alter the optimum phenotype in an ancestral range, as well as leading populations to move into new areas and expand their ranges (Hickling et al., 2006; Parmesan & Yohe, 2003). Increasing anthropogenic disturbance can lead to changes in experienced heterogeneity through new configurations of environments across a landscape (Holyoak & Heath, 2015) that could change the optimal niche breadth of a species, altering whether generalism or specialism is selected for (Chevin et al., 2010). Global change has been shown to trigger both range expansions and increased expression of plasticity, and it is possible that range expansions and increased plasticity can have synergistic impacts (Matesanz et al., 2010). Understanding how the ecological impacts of range expansions and clustering play a role in the evolution of niche breadth can help us predict how species will respond to the challenges they are currently facing.

Random dispersal across a heterogeneous landscape can potentially lead to the evolution of an expanded niche by causing organisms to experience a broad range of environmental conditions. This expanded niche can have a variety of forms: specialization to a favored environment, habitat choice, or generalism (Bradshaw, 1965; Levins, 1968; Scheiner, 2014a, 2014b, 2016; Snell-Rood & Steck, 2018). There are multiple types of generalism, including using multiple environments by producing many variable offspring (bet-hedging), producing a fixed-average phenotype that does moderately well in a range of environments (jack-of-all-trades), or using environmental cues to produce phenotypes that are close to the optimum in their current environment (phenotypic plasticity) (Levins, 1968; Scheiner, 2014a, 2014b, 2016). Experienced heterogeneity is important in determining both whether generalism evolves and what type of generalism arises (Scheiner, 2013, 2014b; Sultan & Spencer, 2002). One overarching prediction is that in landscapes with higher experienced heterogeneity, specialized organisms will often encounter suboptimal environments and generalists will be more favored (Ben-Hur & Kadmon, 2020; Buckling et al., 2007; Gianoli, 2004; Kassen, 2002; Lind & Johansson, 2007; Lind et al., 2010; Scheiner, 1998, 2013, 2014a; Sultan & Spencer, 2002). The way that organisms experience heterogeneity can be shaped by whether differences appear frequently (fine-grained variation) or infrequently (coarse-grained variation), and by whether it is spatial or temporal (Bradshaw, 1965; Levins, 1968). When heterogeneity is spatial, these grain sizes can also be relative to an organism’s dispersal distance, with longer dispersing individuals experiencing the landscape as more fine-grained and encountering more variation (Bradshaw, 1965; Chevin & Lande, 2011; Hollander, 2008; Levins, 1968; Scheiner, 2014a, 2014b, 2016; Scheiner et al., 2012; Snell-Rood & Steck, 2018). Specialized ecotypes can exist on coarse-grained landscapes, where organisms experience less heterogeneity, but fine-grained landscapes can more strongly push populations to evolve generalism (Bradshaw, 1965). All else being equal, we can predict that generalists are more likely to evolve in populations where organisms and their progeny are experiencing more variation in environmental conditions, and grain size is one factor that can alter the amount of that variation (Hollander, 2008; Levins, 1968; Lind et al., 2010; Scheiner et al., 2012).

In addition to impacting experienced heterogeneity, the grain size of clustering can impact range expansions (Chevin & Lande, 2011; Eigentler et al., 2022; Rodewald & Arcese, 2016). Consider a specialist that is well-adapted only to its ancestral environment, $E_{A}$, but is encountering a novel environment, $E_{N}$, as it expands into new territory. In fine-grained landscapes, a high proportion of the specialist’s offspring will disperse to unfavorable environments, but the whole landscape has similar heterogeneity and there are no significant barriers to expansion. Meanwhile, in coarse-grained landscapes, the larger clusters of $E_{A}$ will provide greater benefits to ancestral specialists, but the larger clusters of $E_{N}$ have the potential to completely block the progress of expanding populations (Gralka & Hallatschek, 2019). In coarse-grained landscapes, mutations that enable an individual to subsist in these novel habitats, even if only poorly, can allow it to escape crowding in the ancestral habitats and continue expansion (Baym et al., 2016; Greulich et al., 2012). With further evolution, such mutants could expand into adjacent, uncolonized patches of $E_{A}$, gaining further advantage. In this scenario, coarse-grained landscapes present less heterogeneity, but might offer greater ecological opportunity for the accumulation of mutations that alter the organism’s niche breadth.

These ecological opportunities are particularly relevant when the evolution of niche expansion is constrained. Niche breadth evolution could require multiple mutational steps (e.g., Draghi, 2019), as was empirically demonstrated by Meyer et al. (2012). Constraints can also arise from genetic correlations among traits under selection (e.g., Etterson & Shaw, 2001; see Svensson et al., 2021). These constraints could cause complex traits to evolve in multiple steps, with different selection pressures acting on each step.

In this study, we model the evolution of plasticity as a complex adaptation that requires several mutations to optimize. We quantify how often this adaptation arises in expanding and settled populations with different grain sizes of heterogeneity, seeking to understand how these conditions can impact selection on the entire evolutionary trajectory to plasticity. In settled populations without range expansion, we predict that fine-grained heterogeneity—which more strongly favors plastic generalists over specialists—will accelerate the evolution of plasticity. However, we predict that in expanding populations coarse-grained heterogeneity may favor precursor mutations by rewarding mutants that can exploit an alternative environment. Because these ecological benefits favor mutations that can also lead to plastic generalism, we expect plasticity to be more likely to evolve in coarse-grained landscapes, but only in expanding populations.

Using individual-based simulations on complex landscapes, we confirmed both predictions: in settled populations, plasticity arose most often in fine-grained landscapes while in expanding populations plasticity arose most often in coarse-grained landscapes. To confirm that ecological opportunity was responsible for this boost in evolvability, we also simulated populations expanding either parallel or perpendicular to stripes of alternating environments. Here, populations evolved plasticity most readily when these stripes impaired the expansion of specialists. These results demonstrate a novel interaction between the ecological and evolutionary effects of landscape structure, helping to illuminate how the spatial structure of environments shapes adaptive solutions to heterogeneity.

## Model

### Genotype-phenotype-fitness relationships

Models of the genetic basis of plasticity range in complexity from a few loci with pre-determined trait effects (e.g., Botero et al., 2014; Scheiner, 2013; Tufto, 2015) to complex gene networks (e.g., Draghi & Whitlock, 2012). Because our purpose here is to explore ecological complexity in large populations, we chose a simple model of genotype-phenotype relationships; namely, the equation for a line:


$$\begin{array}{l} z_{i}=a\;c\left(i\right)+b+N\left(0,\;\sigma \right) \end{array}$$ (1)

$z_{i}$ is the organism’s phenotype in their current environment, *i*. Two mutable parameters, *a* and *b*, represent the contributions of plastic and constitutive genes, respectively. The slope, *a,* is multiplied by an environmental cue, *c(i)*, which varies with the environment. Developmental noise is drawn from a normal distribution with a mean of zero and a standard deviation σ = 75, which does not evolve.

Fitness was determined through Gaussian stabilizing selection with distinct optimum trait values, $z_{opt,\;i}$, in each environment.


$$\begin{array}{l} w=exp\left(-\frac{{\left(z_{i}-\;z_{opt,\;i}\right)}^{2}}{2\sigma_{opt}^{2}}\right) \end{array}$$ (2)

The value of $\sigma_{opt}^{2}$ determines the width of the fitness function, or how strongly selection is acting as phenotypes move further from the optimum, and was equal to 7,500 for all simulations presented here. Note that plasticity (*a*) has no direct costs, but affects fitness only via its effects on $z_{i}$.

**Figure 1.**
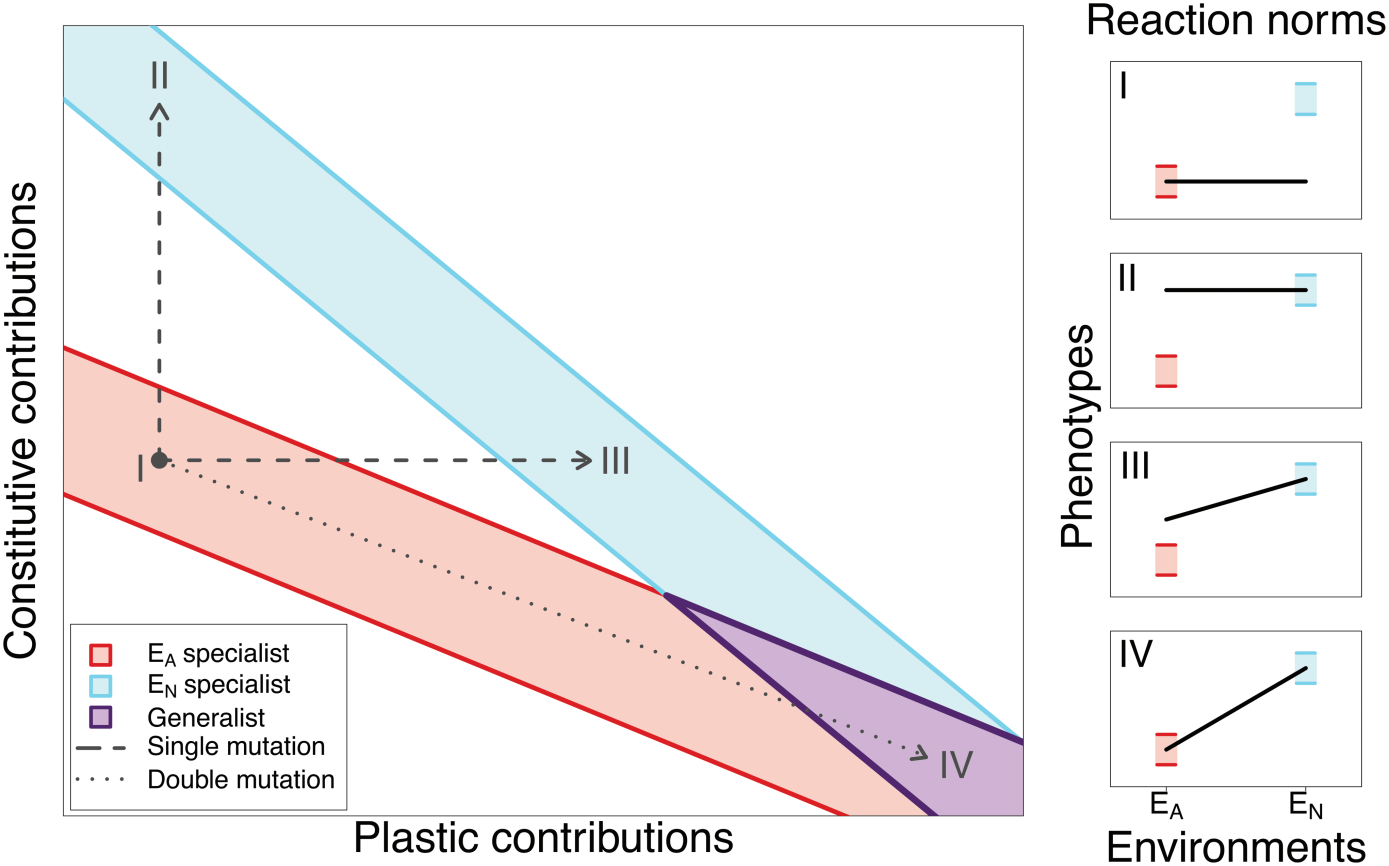
A simplified fitness landscape mapping model genotype parameters *a* and *b* onto niche classifications. Shaded areas between red lines and blue lines indicate genotypes that would be considered a specialist, with an absolute value of at least 1 in the absence of phenotypic noise and competition, in environments $E_{A}$ and $E_{N}$, respectively. Genotypes that would produce a generalist capable of using both environments are shown by the purple shaded area in between purple lines. Single mutations, depicted with dashed lines, change only one genotypic parameter and rare double mutations, depicted with a dotted line, change both genotypic parameters. Reaction norms for points on the fitness landscape, denoted with roman numerals, are plotted along the right side and with colored blocks denoting the optimum in each environment.

We model two environments; , for which starting organisms were optimized and , in which starting organisms had very low fitness. Evolution from the ancestor (*a *= 0, *b* = 1,000) to a perfect plastic generalist (*a* = 1, *b* = 0) requires substantial change in both parameters, which mutate separately. This requirement for multiple mutations creates an evolvability constraint on plastic generalism. Since a non-zero cue is present in both environments, changes in *a* will impact the phenotype in both environments. An increase in the plastic contributions without a complementary decrease in the constitutive contributions will lower the fitness in the ancestral environment (Draghi, 2019). Essentially, the traits across environments are strongly but mutably correlated. These deleterious pleiotropic effects could be mitigated by a simultaneous mutation in constitutive genes (Figure 1), but the probability of both genes mutating at the same time in the right amount to increase fitness is extremely small for realistically low mutation rates. Pleiotropic constraints could also be mitigated by sequential compensatory mutations. Figure 1 shows that single mutations could change an organism from a specialist in one environment to a specialist in the other. A sequence of such changes could potentially lead to a generalist without the need for multiple, simultaneous mutations.

## Methods

The population was simulated using a version of the Muller model on a spatial landscape with *N* square patches, arranged in a lattice with length *L* and width *W.* Each patch was assigned a 0 (for $E_{A}$), or a 1 (for $E_{N}$), and could contain one or zero organisms. The environment of each patch remained constant during a simulation. A generation was operationally defined as follows: first, the addresses of each patch were permuted randomly, then each patch was evaluated in that order. If a patch was occupied, the organism in it experienced the following life cycle. First, its phenotype and fitness were calculated using equations 1 and 2. That organism then asexually produced a Poisson-distributed number of offspring with a mean determined by multiplying its fitness by a fecundity constant; we used a value of 10 for all simulations. Offspring then dispersed to neighboring cells with movement probabilities determined by a discretized two-dimensional Gaussian movement kernel with a mean of 0, a standard deviation of *D*, and zero covariance. Draws from their kernel were conditioned on leaving the natal patch, and if offspring dispersed off the edge of the landscape or into an occupied cell they were lost from the population. After reproduction, the focal organism was removed from the population. Note that generations are overlapping, as in the standard Muller model: an offspring could reproduce in the same generation as it was born depending on the order in which coordinates were evaluated.

At birth, each locus (*a* and *b*) could mutate with the same probability μ—mutations in both were permitted and occurred with probability μ^2^. The value of slope (*a*) mutations was randomly drawn from a normal distribution *N*(0,0.25) and the value of intercept (*b*) mutations was randomly drawn from distribution *N*(0, 500). Mutations were added to the organism’s current gene value. In $E_{A}$, the cue and optimum were both 1,000 while in $E_{N}$ the cue and optimum were both 2,000. These values and equations 1 and 2 are based on Draghi (2019); their magnitudes interact with the strength-of-selection and mutations-effect-size parameters but are otherwise arbitrary. Given these values, an optimal plastic generalist has the genetic values *a* = 1 and *b* = 0. Initial organisms, which are adapted to the ancestral environment and are not plastic, are assigned values *a* = 0 and *b* = 1,000.

We measured the degree of constraint on the evolution of plasticity by the percentage of replicates in which plasticity evolved within a set time frame. A genotype was considered a “plastic generalist” if the product of its fitness and the fecundity parameter (10) was at least 1 in both the ancestral and novel environments, equating to at least 1 expected offspring in both environments. Once at least one plastic generalist had achieved a lineage size of 100 cumulative copies, it was considered established and that replicate was recorded as having successfully evolved plastic generalism.

To create *clustered* landscapes, we first generated a matrix with dimensions *L × W* and alternating patches of $E_{A}$ and $E_{N}$ in a checkerboard pattern. This checkerboard pattern was the starting point for generating all clustered landscapes because it reflected the most fine-grained clustering possible. To increase clustering, we applied the following algorithm (Draghi, 2019). First, the number of pairs of patches to be swapped was determined by multiplying *N* by a clustering parameter, θ. Each of member of the pair was then evaluated for similarity to its immediate neighbors, defined as the eight bordering patches (direct and diagonal neighbors). If both patches were different from the majority of their neighbors and had different environments from each other, then their identities were swapped. This procedure was designed to maintain the prevalence of each environment while increasing the granularity of their distribution. The values of θ used in this experiment ranged from 0.5 to 370, with 0.5 creating the most fine-grained treatment and 370 creating the most coarse-grained treatment (Supplementary Figure 1). We performed between 200 and 600 replicates for treatments in the clustered landscapes.

To quantify the level of clustering, or grain size, in the *clustered* landscapes we used Moran’s *I* as a measure of autocorrelation. Moran’s *I* determines how similar a focal point is to the points around it and then weights the contributions with a matrix of distances ($m_{ij}$) between the focal point and each point evaluated. On our landscapes, this is:


$$\begin{array}{l} I=\;\underset{i=1}{\overset{N}{\sum }}\;\underset{j=1}{\overset{N}{\sum }}\;m_{ij}\;\left(1-{\left(x_{j}-x_{i}\right)}^{2}\right) \end{array}$$ (3)

We generated the *m*_*ij*_ matrix from the dispersal kernel, creating a measure of autocorrelation that was specific to the heterogeneity experienced by our populations.

The two demographic treatments used in the *clustered* landscapes were a *settled* treatment, where the landscape is fully colonized with the starting organism at the beginning of the experiment, and an *expanding* treatment, where the population spreads through the empty landscape during the treatment. In the *settled* treatment, the starting organisms were randomly placed across the landscape with each cell having a 10% chance of being seeded. In the *expanding* treatment, the starting organisms were all placed along the first column (left edge) of the landscape. All of the landscapes in the *clustered* experiment have a height of 250 patches and a width of 650 patches. Aside from the placement of starting individuals, simulations for *settled* and *expanding* treatments were run identically. In this experiment, *D* = 1.58 and the maximum number of generations before simulations were cut off in this experiment was 2,000, which was several times more than was required for expanding populations to reach the distal side of the landscape.

To create *striped* landscapes, we created stripes of alternating environments of varying widths. We used widths ranging from 3 to 40, and used the same maximum number of generations as in the *clustered* treatment, 2,000. In order to resolve more detail on the interaction between the probability of dispersing across the stripes and the time to evolve generalism, we used larger dispersal distances in this experiment with *D* = 3.16. The *striped* landscapes had an *against-the-grain* treatment where the direction of population expansion was perpendicular to the stripe orientation and a *with-the-grain* treatment where the direction of expansion was parallel to the stripe orientation. All treatments in the striped landscapes had 100 replicates.

## Results

**Figure 2.**
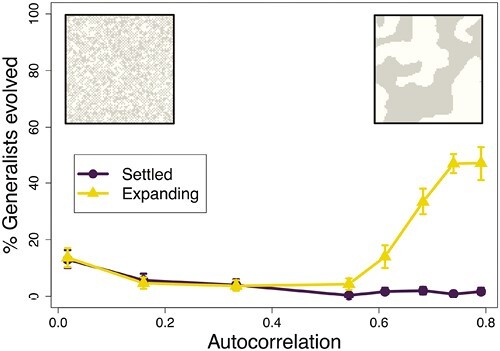
The percentage of replicates where plastic generalism evolved on landscapes with a variety of levels of clustering. Moran’s *I*, was used to quantitatively assess the degree of autocorrelation in different grain sizes. The settled treatment is plotted with circles and the expanding treatment is plotted with triangles. Left inset shows an example of a landscape with fine-grained clustering, right inset shows an example of a landscape with coarse-grained clustering. Parameter values for these simulations are μ (mutation rate) = 0.0005, fecundity = 10, *D* (dispersal kernel distance) = 1.58, *N* (landscape area) = 162,500, and max. generations = 2,000. All treatments had 300 replicates except for the I= 0.61 and I=0.79 expanding landscapes (which had 200 replicates), and the I= 0.74 settled and expanding landscapes (which had 600 replicates). Error bars are bootstrapped 90% confidence intervals.

To evaluate if range expansion alters the relationship between environmental clustering and the evolution of plasticity, we evolved populations on heterogeneous landscapes with varying levels of spatial clustering. We compared two treatments, one in which populations expanded into the landscape from an initial refugium located on one side (*expanding*) and another in which they were initially seeded throughout the landscape (*settled*). In *settled* treatments, the percentage of replicates where plastic generalism evolved decreased with increasing clustering (Figure 2), as predicted by the relationship between experienced heterogeneity and fitness of plastic generalists. In *expanding* treatments, the percentage of replicates where plastic generalism evolved also decreased from fine-grained to intermediate-grained landscapes. However, the propensity for plastic generalism to evolve greatly increased in more coarse-grained landscapes. Out of all the treatments, plastic generalism evolved most readily in the coarsest-grained *expanding* treatment, considerably diverging from predictions based on experienced heterogeneity alone.

**Figure 3.**
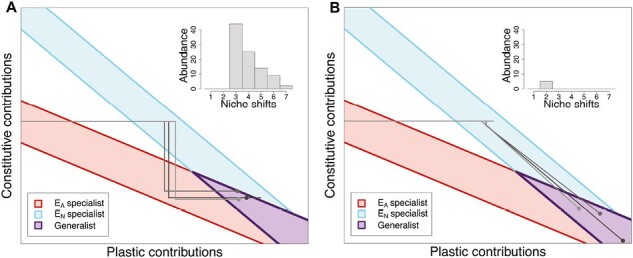
Large graphs show the mutational trajectories of randomly selected lineages that evolved plastic generalism in coarse-grained landscapes in the expanding (A) and settled (B) treatments. The sequence of genotypes that led from the ancestral $E_{A}$ specialist to a generalist are shown with plastic generalist endpoints indicated by a round point. Shaded red areas inside red lines indicate genotypes that are $E_{A}$ specialists, shaded blue areas inside blue lines indicate genotypes that are $E_{N}$ specialists, and the shaded purple area inside purple lines indicate genotypes that are plastic generalists. Inset histograms summarize the number of niche shifts in lineages that evolved generalism in each treatment. The clustering treatment these graphs come from is the most coarse-grained clustering in Figure 2, with I=   0.79.

To understand the divergence in evolutionary dynamics between the *settled* and *expanding* treatment in coarse-grained landscapes, we looked at the mutational history of lineages that evolved generalism in both treatments. In the *expanding* treatment, the evolution of plasticity is typically accomplished through a series of single mutations that iteratively decrease the contributions of the constitutive genes and increase the contributions of the plastic genes (Figure 3A). Meanwhile, in the *settled* treatment, plasticity typically arises via rare, double mutations (Figure 3B). Based on these data, we hypothesized that single mutations that changed habitat specialization relative to the parent were often positively selected in coarse-grained landscapes, specifically because they could expand into new regions. Intermediate single mutations in the lineages that evolved plastic generalism generally gave rise to upwards of 100,000 total individuals over the course of the simulation, suggesting that they were individually beneficial or neutral rather than being deleterious mutations that were surfing the range expansion front. We also quantified this hypothesis by calculating the number of niche shifts (changes from a $E_{A}$ specialist to a $E_{N}$ specialist, from a specialist to a generalist, etc.) along the evolutionary trajectories that led to plastic generalism. In the coarse-grained *expanding* treatments, most lineages evolved plastic generalism after three or more niche shifts (Figure 3A inset), while in the *settled* coarse-grained landscapes, all lineages evolved plastic generalism in one or two niche shifts (Figure 3B inset). Meanwhile, in the fine-grained treatments, there was a mixture of lineages that evolved generalism through combinations of rare double mutations and through a series of common single mutations in both expansion treatments (Supplementary Figure 2). These results indicate that in coarse-grained landscapes, range expansion influences not only the speed but also the path of evolution. To better understand the importance of niche shifts allowing continued expansion, we next examined landscapes with well-defined barriers to expansion.

**Figure 4.**
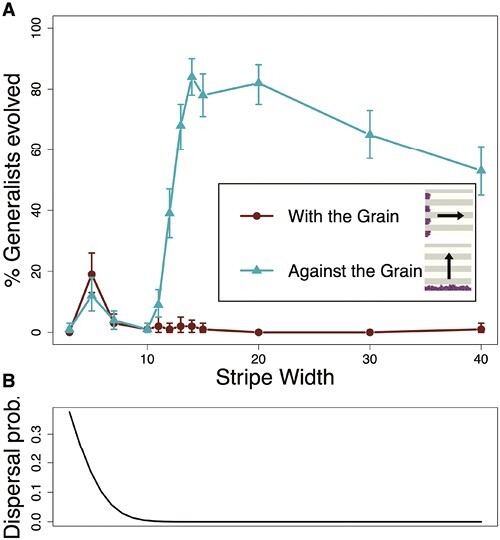
(A) The percentage of replicates that evolved generalists in the striped experiment on with-the-grain replicates (shown with circles) and against-the-grain replicates (shown with triangles) at different stripe widths. Inset figures in the legend give an example of what landscapes for each of the treatments look like once range expansion has started with arrows indicating direction of expansion. Parameter values for these simulations are μ (mutation rate) = 0.0005, fecundity = 10, *D* (dispersal kernel distance) = 3.12, *N* (landscape area) = 160,000, and max. generations = 2,000. All treatments in this experiment had 100 replicates. (B) The probability of an organism dispersing across a stripe at different stripe widths. As the stripe width increases, the probability of dispersing across it decreases. Error bars are 90% bootstrapped confidence intervals.

To specifically investigate how coarse-grained structure acts to restrict expansion, we designed landscapes that had alternating stripes of $E_{A}$ and $E_{N}$ with variable stripe widths. With these *striped* landscapes, we had two treatments: a *with-the-grain* treatment, where the direction of range expansion was parallel to the stripes, and an *against-the-grain* treatment, where the direction of range expansion was perpendicular to the stripes (Figure 4A in legend). In the *with-the-grain* treatment, $E_{A}$ specialists were able to reach the opposite side of the landscape without needing to pass through regions of $E_{N}$. Meanwhile, in the *against-the-grain* treatment, $E_{A}$ specialists had to pass through successive, alternating blocks of $E_{A}$ and $E_{N}$ before reaching the opposite side of the landscape. This allowed us to vary whether clustering was an explicit barrier to range expansion while holding the experienced heterogeneity and presence of range expansion constant.

At lower stripe widths, *against-the-grain* and *with-the-grain* treatments were similar (Figure 4A). In the *with-the-grain* treatment, the percentage of replicates in which plastic generalism evolved was very low and decreased further with increasing stripe widths, similar to the trend in the *settled* landscapes of the *clustered* treatments. In the *against-the-grain* treatment, the percentage of replicates where plastic generalism evolved abruptly increased at stripe widths between 10 and 15, and then slowly decreased at increasing stripe widths. This spike correlates with the dispersal kernel in this treatment (Figure 4B); once stripes are too wide to jump over through dispersal and organisms need to move through them, the percentage of replicates where generalism evolves dramatically increases. This supports our hypothesis that coarse-grained heterogeneity stimulates the evolution of generalists specifically by presenting barriers to the expansion of specialists.

## Discussion

Our results show that the landscape where generalists have the highest relative fitness—fine-grained clustering—is not always the most conducive to their evolution. Instead, coarse-grained landscapes stimulated the evolution of generalists during expansions by blocking the movement of specialists and providing ecological opportunities to niche-shifting mutations during range expansion. This selective environment facilitated the exploration of genotype space, allowing generalism to evolve via relatively long sequences of single mutations. Here, range expansion and the landscape structure interact, allowing a population to evolve a complex phenotype without the need for rare, simultaneous mutations. These results suggest that predicting the emergence of evolutionary innovations depends on demographic considerations, such as the history of movement through the landscape. For example, in the striped experiment (Figure 4), the direction from which populations approached a new landscape predicted whether generalists would emerge. Only modeling evolution in an already-colonized landscape would miss this key distinction. Transient demographic factors can therefore alter which evolutionary trajectories are taken.

Range expansions are one factor that can have dramatic eco-evolutionary feedbacks, here facilitating the evolution of new niche breadth strategies even in the face of constraints. Another consequence of range expansions is an amplified impact of stochastic forces on genotype frequencies (Edmonds et al., 2003; Gralka & Hallatschek, 2019; Hallatschek et al., 2007; Lambrinos, 2004; Miller et al., 2020). A specific pattern of stochasticity being amplified is allele surfing, where mutations that occur on the front of an expanding wave reach higher frequencies than would be expected from fitness and genetic drift alone (Edmonds et al., 2003; Excoffier & Ray, 2008; Hallatschek et al., 2007). In this study, patterns of mobility through landscapes seemed to be more important to evolutionary outcomes than allele surfing, as seen by the difference in replicates that evolved plastic generalism in the *striped* experiment, where both treatments involved range expansion. These results demonstrate range expansion can ecologically alter the amount of heterogeneity an organism experiences and change how broad of a niche is favorable.

Range expansions also create the potential for organisms to be exposed to novel environments. In our model, organisms dispersed randomly, without regard to the type of habitat they were crossing. However, in nature organisms vary in their propensities to cross gaps of unfavorable habitat. The size of clusters of unfavorable habitats, detour efficiency, and trade-offs in animal life history can alter how likely animals are to cross these gaps (Bakker & Van Vuren, 2004; Duggan et al., 2012; Silva et al., 2020; Snyder et al., 2022). Mutations can allow organisms to use novel environments; even mutations that only allow poor use of a new environment could be favored if they can spread quickly in the new unoccupied areas (Baym et al., 2016). Past work has suggested that plasticity is one way for populations to quickly respond to new environmental conditions (Diamond & Martin, 2021; Scheiner & Levis, 2021). This has led to speculation about how common greater plasticity is in expanding populations, but Lande (2016) demonstrated that the amount of plasticity expanding populations produce is contingent on past experienced heterogeneity rather than universally high. In this study, we start with one genotype with zero plasticity, so here there is no cryptic variation in plasticity that new environments can expose. Rather, plasticity can arise as a novel adaptation to both the new environment and the increased spatial heterogeneity.

Past work on phenotypic plasticity has demonstrated complexity in the factors that lead to or constrain the evolution of plastic generalism. Frequency and range of heterogeneity, reliability of cues, and physiological constraints can determine what strategies dominate in any given conditions (Joschinski & Bonte, 2021; Leung et al., 2020; Levins, 1968; Murren et al., 2015; Scheiner, 2014a, 2014b; 2016). In some scenarios, multiple factors could be relevant at once. For example, movement around a landscape can lead to greater heterogeneity (Scheiner et al., 2012) and exposure to more extreme habitats (Chevin & Lande, 2011), both favoring the evolution of plasticity, but it can also decrease the reliability of developmental cues for plasticity and limit the fitness benefits plasticity can provide (Scheiner, 2014b; Scheiner et al., 2012). Our model focuses on irreversible, developmental plasticity. Other models consider plasticity on other time scales such as behavioral or intergenerational (reviewed in Chenard & Duckworth, 2021 and Bonduriansky, 2021, respectively). The specific mechanism of plasticity may be particularly relevant when environments change quickly and a lag between cue perception and response would be deleterious (e.g., Gomes & Cardoso, 2020). In stable environments, such as those modeled here, we would not expect that this aspect of plasticity will play a large role. Future work that added temporal heterogeneity to our modeling framework could consider how our results might change for these different forms of plasticity.

Costs and other constraints on the evolution of plastic generalism have been the subject of much empirical and theoretical work, given that plasticity seems like an ideal, yet relatively uncommon, strategy by which organisms could deal with increased environmental heterogeneity. Previous work exploring the evolution of plasticity has found only weak explicit costs (Buckling et al., 2007; Kassen, 2002; Lind & Johansson, 2009; Manenti et al., 2015; Murren et al., 2015; Snell-Rood & Ehlman, 2021; Van Buskirk & Steiner, 2009). Other potential costs and constraints include limits on how quickly new phenotypes could be produced (Gomes & Cardoso, 2020; Levins, 1968; Siljestam & Östman, 2017) and unreliability of environmental cues (Draghi, 2023; Joschinski & Bonte, 2021; Leung et al., 2020; Levins, 1968). As in our model, plastic generalism may be a superior phenotype to deal with heterogeneity once evolved, but could experience constraints that prevent its evolution (Manenti et al., 2015).

One possible constraint on the evolution of plastic generalism, or other complex niche-widening traits, is pleiotropy. Increasing sensitivity to environmental inputs could increase developmental noise (Scheiner, 2014b) or lead to phenotypic changes in other environments and diverse impacts on fitness. For example, evolving sensitivity to a cue that is present in both ancestral and novel environments could move the phenotype of an organism already well adapted to an ancestral environment away from the optimum in that environment. In this model, we incorporate that constraint by using non-zero cues in all environments (as in Draghi, 2019). This type of complexity constraint, where multiple mutations are necessary before the benefits of a complex adaptation become salient, may be a common barrier to niche expansion. The possibility of multiple mutations needing to be present in concert for a complex adaptation to lead to an emergent fitness increase has been empirically demonstrated with both apparently neutral (Meyer et al., 2012) and individually beneficial mutations (Copley, 2000; Quandt et al., 2015). Even in cases where a fitness benefit is immediately apparent, subsequent mutations can decrease trade-offs and increase fitness across environments, refining the adaptation (Blount et al., 2008; Melnyk et al., 2017; Nagaev et al., 2001; Quandt et al., 2015). Understanding constraints like pleiotropy can help us better predict how plasticity could be part of an organism’s response to new environmental changes and challenges.

This study demonstrates that the factors that select for early steps toward a complex adaptation may differ from the conditions where the adaptation is most favorable. These results point to a limitation of optimality-based models: to predict where we expect to see adaptive plasticity, we need to integrate predictions about the fate of early mutants arising in non-plastic ancestors with an understanding of plasticity’s ultimate benefits. Understanding how the ecology of range expansions and clustering plays a role in the evolution of niche breadth can help us understand how species will respond to the challenges they are currently facing and better plan for the future.

## Supplementary Material

qrad062_suppl_Supplementary_Figures_1-2

## Data Availability

The data and code used in simulations and figure creation for this study are published in a Data Dryad repository (https://doi.org/10.5061/dryad.p8cz8w9xd).
