# Supplementary material for: Range expansion can promote the evolution of plastic generalism in coarse-grained landscapes
Source: Evol Lett. 2023 Dec 14;8(2):322–30. doi: 10.1093/evlett/qrad062 (PMC10959476; doi:10.1093/evlett/qrad062)
Supplement: qrad062_suppl_Supplementary_Figures_1-2 [file qrad062_suppl_supplementary_figures_1-2.pdf]

Supplementary Materials

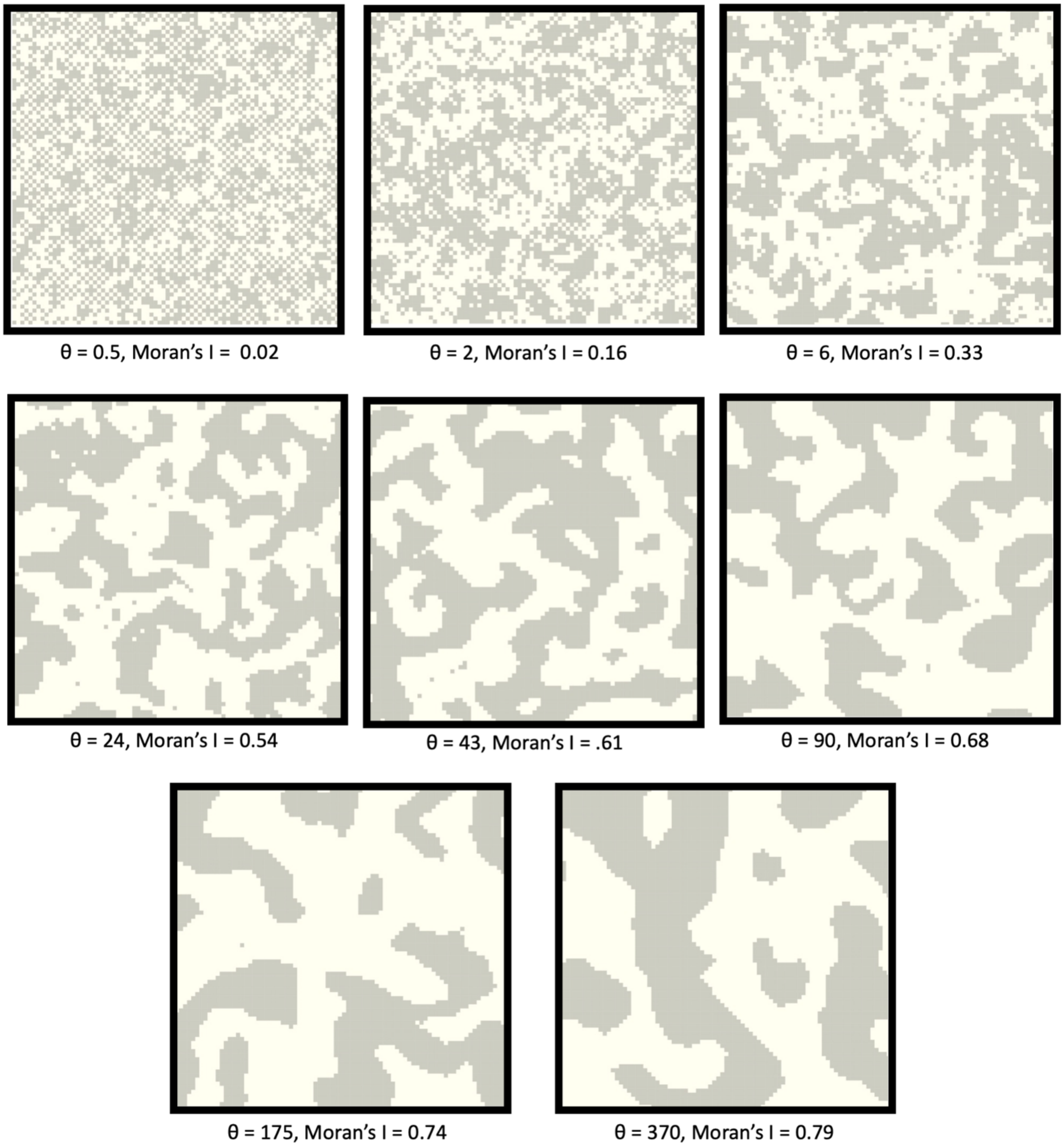

Supplementary figure 1: Images of landscapes generated for the clustered treatment across all values of θ used. Values of θ and Moran’s I associated with each landscape are listed underneath the individual images. Images are a 75 by 75 cell patch from a landscape with the same dimensions as the *clustered* experiment, H = 250 and W = 650.

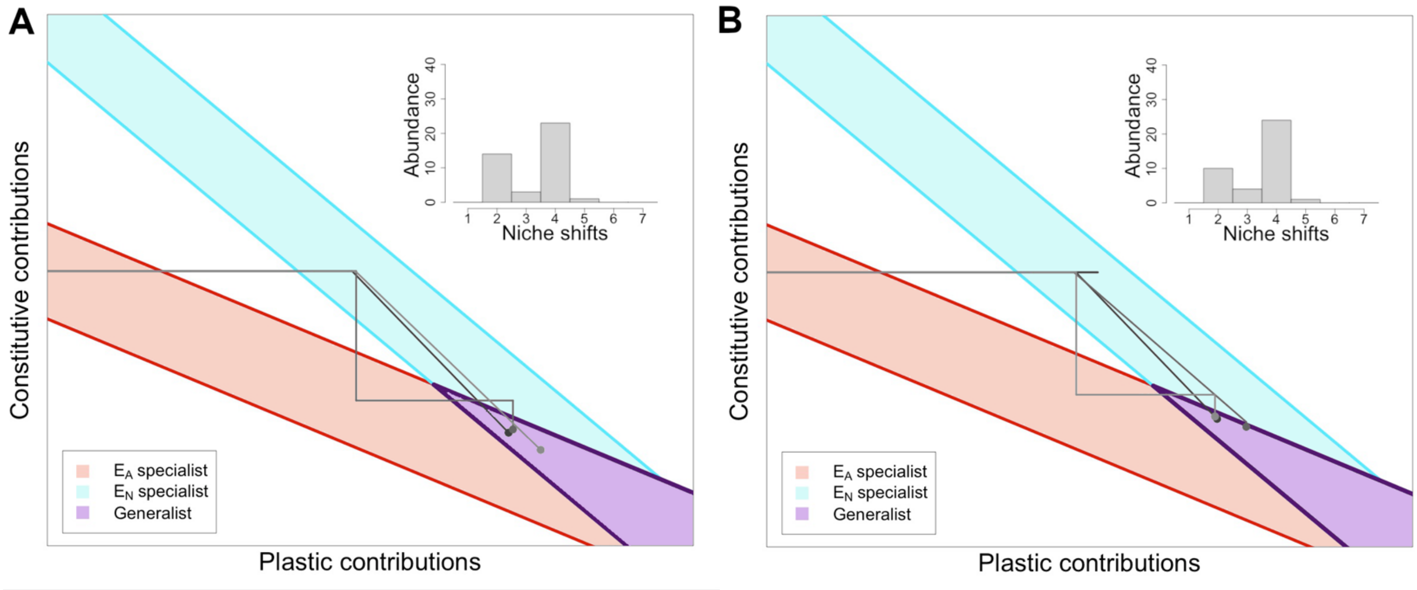

Supplementary figure 2: Large graphs show the mutational trajectories of randomly selected lineages that evolved plastic generalism in fine-grained landscapes in the expanding (A) and settled (B) treatments. The sequence of genotypes that led from the E_A specialist to a generalist are shown with the plastic generalist endpoints indicated by a round point. Red lines indicate genotypes where $W_A = 1$, blue lines indicated genotypes where $W_N = 1$, and purple lines indicate genotypes where $W_A = W_N = 1$. Areas between pairs of lines of the same color indicate genotypes with absolute fitnesses greater than or equal to 1. Inset historgrams summarize the number of niche shifts all lineages that evolved generalism in each treatment experienced while evolving from an ancestral specialist to a plastic generalist. The clustering treatment these graphs come from is the most fine-grained clustering in Figure 2, with $I = .019$.
